# Securing IoT Sensors Using Sharding-Based Blockchain Network Technology Integration: A Systematic Review

**DOI:** 10.3390/s25030807

**Published:** 2025-01-29

**Authors:** Ammad Aslam, Octavian Postolache, Sancho Oliveira, José Dias Pereira

**Affiliations:** 1 Department of Information Sciences and Technologies, University Institute of Lisbon, Av. das Forças Armadas, 1649-026 Lisbon, Portugal; ammad_aslam@iscte-iul.pt (A.A.); octavian.adrian.postolache@iscte-iul.pt (O.P.); sancho.oliveira@iscte-iul.pt (S.O.); 2 Instituto de Telecomunicações, Av. Rovisco Pais, 1049-001 Lisbon, Portugal; 3 Instituto Politécnico de Setúbal, Escola Superior de Tecnologia de Setúbal, Campus do IPS—Estefanilha, 2910-761 Setúbal, Portugal

**Keywords:** sharding-based blockchain technology, IoT sensors, nodes, transactions

## Abstract

Sharding is an emerging blockchain technology that is used extensively in several fields such as finance, reputation systems, the IoT, and others because of its ability to secure and increase the number of transactions every second. In sharding-based technology, the blockchain is divided into several sub-chains, also known as shards, that enhance the network throughput. This paper aims to examine the impact of integrating sharding-based blockchain network technology in securing IoT sensors, which is further used for environmental monitoring. In this paper, the idea of integrating sharding-based blockchain technology is proposed, along with its advantages and disadvantages, by conducting a systematic literature review of studies based on sharding-based blockchain technology in recent years. Based on the research findings, sharding-based technology is beneficial in securing IoT systems by improving security, access, and transaction rates. The findings also suggest several issues, such as cross-shard transactions, synchronization issues, and the concentration of stakes. With an increased focus on showcasing the important trade-offs, this paper also offers several recommendations for further research on the implementation of blockchain network technology for securing IoT sensors with applications in environment monitoring. These valuable insights are further effective in facilitating informed decisions while integrating sharding-based technology in developing more secure and efficient decentralized networks for internet data centers (IDCs), and monitoring the environment by picking out key points of the data.

## 1. Introduction

**Figure 1:**
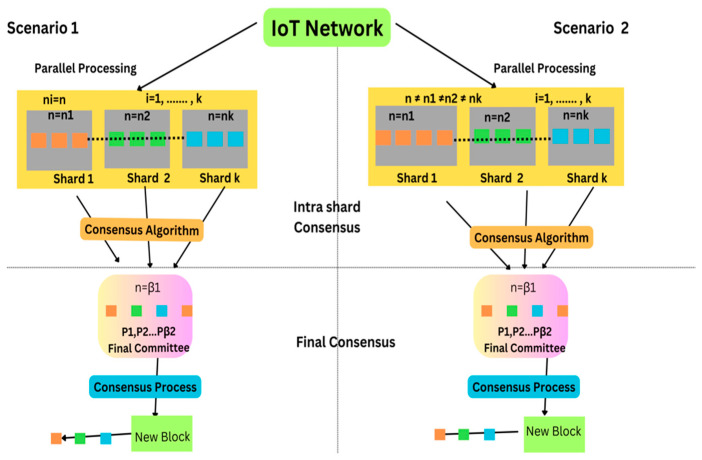
System structure of a sharding-based blockchain network; in Scenario 1, processors are equally distributed among shards, and in Scenario 2, processors are not equally distributed among shards.

The increased adoption of the IoT has made it an appealing technology, which has been adopted within various businesses, industries, healthcare providers, and other departments. The IoT has significantly focused on gathering and exchanging data [1]. Similarly, IoT sensors are also helpful in environmental monitoring as they work by gathering data and measurements from the physical environment by utilizing connected devices and sensors. With the help of these sensors, temperature, moisture, leaks, water levels and other physical properties of tanks, pipelines, industrial equipment, oceanic applications, and irrigation systems can be determined. Furthermore, blockchain technology is considered one of the advanced mechanisms that enables the sharing of information transparently, particularly within a specific business network [2]. With the help of the IoT, devices across the internet are capable of sending data to blockchain network technology, which significantly results in creating tampered-resistant records of all the shared transactions. This is considered important in blockchain network technology, as it involves a distributed ledger system that records all the transactions securely and transparently. Moreover, the sent data are stored and encrypted in an unchangeable manner so that they cannot be manipulated, ensuring the transaction’s security and integrity and making it tamper-resistant. Significantly, sharding-based blockchain network technology, which helps enable scalability in blockchain and other processing technologies, increases the number of transactions every second [3]. This mainly focuses on splitting the blockchain network into shards; however, some other divisions, such as chains, clusters, trees, and graphs, also exist within sharding-based blockchain technology. These shards are significantly responsible for the transaction process of each subset, which further aids in enhancing the efficiency of the blockchain network by enabling the various transactions that are performed simultaneously across different shards, as shown in Figure 1. Sharding is a significant technique that helps enhance the scalability and performance of blockchain technology [4] with the help of a consensus algorithm, as shown in Figure 1.

For example, in Scenario 1, shards are distributed equally, assuming 100 nodes, 10 transactions\second\node, 1 s block time, and 10 shards within 10 nodes, indicating 1000 transaction\second\shard, while in Scenario 2, assuming 100 nodes, 10 transactions\second\node, and 1 s block time, this indicates 1000 transactions\second. Hence, choosing sharding-based blockchain technology is suitable for the IoT environment as it enables the transaction process to be more scalable and suitable at the same time for the IoT environment. Further, IoT devices are operated through computational and battery resources, which helps in energy efficiency. Additionally, this technology is also selected as it provides features like increased security, flexibility, and interoperability. This approach is suitable in the IoT environment as these devices are heterogeneous in nature, and help in real-time data processing, security, and flexibility.

The paper presents a systematic review that further focuses on determining the significant benefits and limitations of sharding-based blockchain network technologies in securing IoT sensors. Also, the study deeply analyses previous studies on the integration of sharding-based blockchain technologies in securing IoT sensors, with a focus on wireless sensor networks (WSN). Furthermore, the organization of the paper can be structured into five sections, where Section 1 is the introduction, which details the significance of the study, the research aim, and the research questions. Section 2 is the methodology section, which is divided into three subsections, involving a systematic literature review, an article search and selection, and a thematic analysis. Section 3 analyses the results of the literature review, exploring the impact, benefits, and challenges of sharding-based technology and monitoring environments. The next section, Section 4, involves the discussion of the results, which is again divided into three subsections based on the three themes. Lastly, Section 5 involves the conclusion and involves one subsection, namely future research directions.

### 1.1. Significance of Study

As discussed above, sharding-based blockchain network technology has been considered significant as it is considered effective in securing the blockchain network technology. This technology ensures that all data are shared effectively with all the significant nodes [5]. Furthermore, these allocated nodes focus on managing the workload of all the shards and significantly minimizing the workload of others, which later on reduces the transaction fee and significantly improves the work efficiency of the blockchain network [6]. This significantly distributes all the workload systematically, ensuring that all the allocated nodes have significant information, leaving room for scalability and also minimizing the cost of transactions on the blockchain network technology [7]. Furthermore, IoT sensors are considered significant for the collection of data within the nearby environment. These IoT sensors are directly or indirectly connected to the IoT networks after signal processing and conversion.

### 1.2. Research Aims, Objectives, and Questions

The present approach focuses on:

- The presentation of the potential of sharding-based blockchain technology for environmental monitoring.
- The determination of the benefits of sharding-based blockchain network technology in securing IoT sensors.
- The examination of the complexities in the existing literature during the implementation of sharding-based blockchain network technology in securing IoT sensors.

Several research questions are considered in the present approach, such as:

➢ What are the potential impacts of sharding-based blockchain technology on securing IoT sensors?
➢ What are the significant advantages of sharding-based blockchain network technologies?
➢ What have previous studies determined about the disadvantages of the integration of sharding-based blockchain network technology?

## 2. Materials and Methods

### 2.1. Systematic Literature Review

A systematic literature review refers to a method that helps in determining, selecting, and critically appraising the study to answer the research questions [8]. It also helps minimize research gaps to provide a clear knowledge of the subject area [9]. As it adopts a well-defined strategy and appropriate planning, it effectively focuses on answering the research questions [10].

With the growing advent of technology, blockchain network technology has also become a huge, significant area of research [11]. Hence, a systematic literature review is also used in the paper to assess the significant challenges and benefits, along with the potential impacts, that accompany the integration of sharding-based blockchain technology.

### 2.2. Article Search and Selection

The selection of articles is one of the most important procedures that helps in identifying and selecting appropriate articles that focus on contributing to the research questions [12]. This further focuses effectively on publications based on sharding-based blockchain network technology. It also involves inclusion and exclusion criteria, which are considered important as they enhance the validity of the study and its feasibility, and also lessen its cost [13]. Furthermore, it identifies relevant and high-quality publications by assessing their suitability through their abstract, full-text, journal name, and publication year.

#### 2.2.1. Database Search

The selection of articles was carried out using MDPI and IEEE Xplore. Being a new and popular exploration field, the search strategy used in this study was not very restrictive, which ensures the study’s reliability, as it only involves a few publications [14]. Further, reflecting on the expected time to complete the research, the search was limited to 2022 to 2024, which ensured that the research involved the most recent literature reviews that were covered in-depth. The search terms also involved the use of Boolean operators, like (and OR), which enabled the researcher to try different combinations for the search. Using these search terms helps in deriving specific information on the research topic. Further, to find appropriate studies, it is significant to determine unique search options to determine the wide variety of materials available for the study. Also, the use of Google Dictionary and Thesaurus is of great help to find out correct synonyms of the important words in the research questions. This effectively helps in identifying relevant search terms in Google Scholar. Further, the databases were searched using the Boolean operators and the search terms used were “IoT sensors AND sharding-based blockchain”, “Benefits OR challenges of Sharding-based technology”, and “Integration of sharding-based blockchain AND its impact on IoT sensors”. The keywords of the search were “Sharding blockchain technology”, “IoT sensors”, “Environment”, “Healthcare”, and “Agriculture”.

#### 2.2.2. Inclusion and Exclusion Criteria

Inclusion criteria are defined as the characteristics that determine whether publications are be included in the study, while the exclusion criteria are defined as characteristics that concentrate on disqualifying prospective publications from study inclusion [15]. The study involved appropriate inclusion and exclusion criteria, which ensured the data quality of the studies that were included in the analysis. Publications with complete, reliable, and trustworthy information were included in the study. The study excluded publications with incomplete information that were published before 2022. Exclusion of articles was performed by assessing the publications’ information and keywords. The researcher first focused on ensuring the significant information about the publication (including the title, author’s name, publishing journal name, and date of publication) and determined if the information was true and complete to ensure its trustworthiness and reliability [16].

#### 2.2.3. Overview of Publications

The researcher further focuses on familiarizing with the research questions mentioned in the publications to ensure that this literature fits perfectly in the study and is credible and significant [17]. The aim focuses on ensuring that the publications scrutinize the integration of sharding-based blockchain network technology by collecting real-time information for the control, monitoring, and automation of the connected systems by measuring their pressure, motion, and temperature. While familiarizing themselves with the key aspects of the included publications throughout the process, the researcher categorized the included publications based on the publication type (book, conference paper, journal article), and contribution type (theoretical, or empirical).

### 2.3. Classification of Articles

The systematic literature review offers an overview of the study by thoroughly determining and explaining the significant impacts, benefits, and complexities of the emerging technology. Some of the explored articles are as follows:

- The potential impact of sharding-based blockchain technology in securing IoT sensors: Papers related to environmental monitoring, with an increased focus on sharding-based blockchain technology in the field of IoT sensors have been explored. It further involves how it affects the environment by determining the significant advantages of its implementation.
- The benefits of implementing sharding-based blockchain technology in securing IoT sensors: Various articles have been explored to determine the benefits of sharding-based technology by effectively identifying the significant effects of this technology in various fields.
- The complexities in integrating sharding-based blockchain technology to secure IoT sensors: Studies have explored the difficulties faced in the integration of this emerging technology. Further, this would also help in understanding the positive and negative effects by incorporating sharding-based blockchain technology in different fields, such as agriculture, healthcare, and others.

### 2.4. Thematic Analysis

The researcher has further conducted a systematic literature review with the help of thematic analysis. It refers to the primary qualitative method used in the research [18]. It is an exploratory process that gives a deep understanding of the topic by exploring and identifying significant patterns of data and organizing them into themes in a hierarchical manner [19]. In this study, thematic analysis focuses on facilitating and systematically determining the benefits and complexities of blockchain network technology integration and securing IoT sensors to understand the similarities across different publications included in the systematic literature review.

It involves six steps, including the familiarization of data, the generation of the initial code, searching and reviewing themes, defining themes, and the final write-up [20]. Following these steps helps in systematically reviewing the publications according to the research questions by identifying significant themes (see Table 1).

## 3. Results of Literature Review

### 3.1. Impact of Sharding-Based Blockchain Network Technology on Securing IoT Sensors in Environmental Monitoring

Sharding-based blockchain technologies have gained increased attention to address environmental issues. Additionally, it also provides several opportunities for managing environmental risks. The emphasis of this section is on understanding the effect of sharding-based blockchain technology in securing IoT sensors, and mostly determined positive impacts; however, it also determined several issues in the technology. The following literature has demonstrated several key findings.

**Figure 2:**
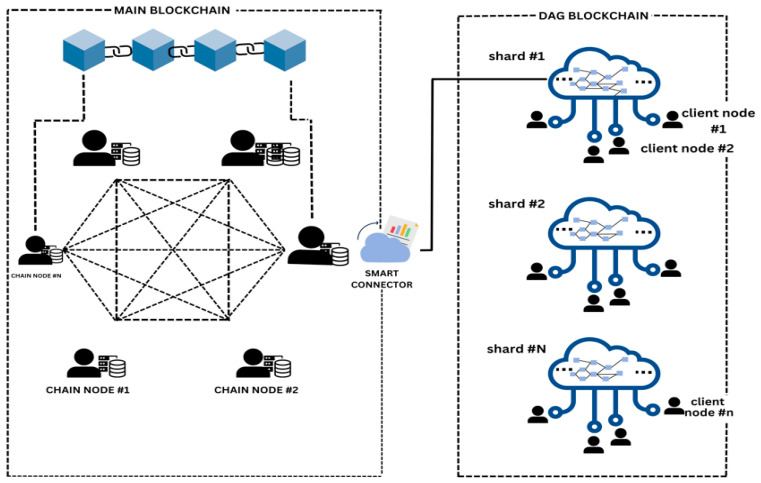
DAG protocol system architecture [26].

The results of [21] determined the positive impact of blockchain technology, which offers a feasible solution, with a focus on reducing implementation costs and maintenance. It also demonstrates the SDR-based low-cost receivers that help in minimizing the scalability attrition, while ref. [22] determines the drawbacks of blockchain technology within the IoT domain. It determines the security and scalability of the firmware protocols. Further, ref. [23] determines the positive impacts of blockchain in the IoT, offering inefficiency in peer communication to be addressed through flooding techniques to share blocks that aid in inefficient and duplicity bandwidth usage. Similarly, ref. [24] also explored the positive impact of the IoT with blockchain technology by assessing the security of the blockchain network technology to ensure its availability, integrity, and confidentiality. Further, the results of [25] show improved storage efficiency offering significant insights into the effectiveness of increased long-term sustainability analysis. Furthermore, ref. [22] also demonstrates efficiency and reliability, as the authors indicate secure development and scalable updates in the system. However, this system significantly lacks validation in blockchain technology’s automotive OTA system updates, which requires decentralization. Also, ref. [26] explores adaptable and versatile simulators, which have been considered an effective evolution in blockchain technology. However, it also determines a lack of public discrete event simulators that can result in determining the distributed ledgers based on DAG, as shown in Figure 2, which would help in designing a scalable and secure way of conducting transactions and managing data through a decentralization process. It is mainly a distributed ledger, used to record transactions and achieve consensus by utilizing the graph data structure within the network.

The study of [27] explores an aggregated learning process that offers secure updates on global models and shows potential performance bottlenecks. From the study of [28], improved intra-shard transactions, which improve the security of blockchain technologies, resulting in the enhanced security of securing IoT sensors, are determined. This was performed through a consistent hash algorithm, along with a signature Anchorhash that aids in reducing the mapping of the node assignment, which further improves the significant procedure of cross-shard transaction, and also uses the nodes’ activity for the reconfiguration of nodes. Additionally, ref. [29] helps decrease the delays of system service by guiding the nodes to cache the entire blockchain system across the particular network. However, ref. [30] opined an increased usage of the IoT, which offers internet connectivity and digital connectivity with the help of sharding-based blockchain technology, that results in high productivity and helps determine the countermeasures against cyberattacks. As depicted by [31], unsupervised learning, and unsupervised learning-based anomaly detection in IoT systems, show integrity and security issues within the blockchain networks. With this, a mutual relationship between anomaly detection and unsupervised learning is determined, which reviews the algorithm categorization issues, which is utilized in the study as an implementation and integration process. It further reveals that the characteristics of the three categories are effectively determined in the manner that determines how the implementation process takes place. While ref. [27] significantly demonstrated learning process aids in preventing model-poisoning attacks and effectively determines a significant and secure update of the global model. This significant knowledge helps acquire an enhanced understanding of the sharding-based blockchain network systems. The study of [32] assessed the redesign of the communication networks along with storage architectures and distributed computing, which aids in maximizing the cost or performance ratio that is further effective in meeting the demanding complexities of flexibility, availability, scalability, bandwidth, and latency. Significantly, ref. [24] explained with the help of secure group communication, along with fully homomorphic encryption with optimal essential generation technique, that it is effective in the IoT environment through data routing and encryption. While ref. [29] assessed the overall performance of the network technology. With the help of the content sharding method, caching can be optimized effectively, and it also results in improving the cache-hitting ratio. The results of [33] explain that in the production of unchangeable logs of transactions within these blockchain networks. Further, as investigated by [34], the increased integration of the IoMT has played a significant role in offering its citizens a better quality of life, offering significant benefits to them.

As per the findings of [35], blockchain technology helps secure data and prevent them from being forged. It also shows an increased demand for data scaling. The findings further visualize a technique that helps in storing data with the availability and scalability of the blockchain network by maintaining its security, with the help of VOSviewer and CiteSpace. As explained by [36], the integration of blockchain technology plays an important role, as it helps in determining the technological infrastructure of food security. Initially, the food supply chain experienced several untraceable and inefficient corruptions, which led to the integration of blockchain technology. The different pillars of food security and the significant implementation of the technology’s management, organizational, and economic aspects are also understood. Further, in [35], these significant benefits of this technology have resulted in determining its role in the integration of IoT applications. The significant key aspects are the availability, scalability, and storage of the blockchain technology. Further, the study of [37] explains that integrating blockchain helps improve sustainable development in the agriculture sector. The significant use of blockchain technology effectively enhances the robustness of the classification responses that were demonstrated during plant diseases. Another study by [33] explains that blockchain technology helps in effectively securing and exchanging data, by producing unchangeable logs of transactions and effectively communicating with other IoT nodes. The study by [38] explained that blockchain technology helps address the problem by artificially partitioning the blockchain network into various shards and significant transaction processes while running the consensus of every shard within a subset of blockchain nodes. Moreover, ref. [33] also explained that it helps send data to blockchain technology private networks that further create immutable records of all the important transaction history. Further, ref. [39] determined its increased performance. It is an effective technology that demonstrates the potentiality of AI and blockchain networks that further help in the IDS transformation process.

As determined by [40] the significant role of IoT management in blockchain nodes is maintained through edge nodes, which results in vulnerabilities and determines that vulnerabilities and reliabilities in the blockchain system in securing the IoT cannot be guaranteed, affecting its performance. However, these can be ensured by storing sensor data. Another study by [41] explained that the integration of this sharding-based blockchain technology helps offer the IoT significant effective solutions to deal with privacy and security issues during the integration process. Further, ref. [24] demonstrated that the technique is helpful in employing a trust model which effectively improves the security of the communication process. According to the findings of [42], blockchain is vulnerable to attacks of DDoS, which might result in failures of hardware devices with an increased temperature of 90 degrees Celsius, a significant fire hazard. Moreover, the results also indicated an increased percentage loss of network block and BTR because of corrupted hardware. The results of [43] involve computing nodes to validate the digital transactions and also record the transactions within a unique storage system. It plays a significant role in each node by maintaining a copy of its ledger and validating the transactions by integrating IoT sensors. Additionally, ref. [44] explains that the multi-level blockchain incorporates a chain layer in the IoT devices, which stores the data to ensure reliability. As determined by [45], the scalability feature makes the implementation process easy, as it is the most significant aspect of blockchain technology. The findings of the study have focused on drawing conclusions based on blockchain applications by focusing on different industries, like supply chain, businesses, information management, healthcare, and privacy, by determining their significant challenges. In addition, the study by [22] also determines that stability and performance are not effectively compromised. As propounded by [42], maintaining security, trustless authentication, and resilience are important for the sharing the sensitive data of users in a secure way across the different gadgets of the IoT. The study by [46] determined that scalability is one of the important functions that help improve network technology storage, by proposing a DHT-based blockchain dual-sharding mechanism for the extension of storage. The blockchain network involved m DHT clusters, including n nodes, and storing 1/m of the overall transaction data. Further, the findings of [47] have determined that blockchain technology is effective in acquiring skills, content, and structure to implement learning nodes. It is also helpful in documentation validation. These are inclusive processes that connect blockchain to sustainability and the educational environment, offering a more decentralized system, providing security and integrity, and thus increasing the transaction rate. Lastly, the study by [48] determined that characteristics of security, which effectively focus on decentralization, consensus, and cryptography, significantly aid in ensuring its transaction process by covering the applications in the IoT. Similarly, the study of [49] determined the security of blockchain networks by assessing the impact of performance by proposing the consensus protocol in the IoT. Table 2 and Table 3 present a summary of studies on the impact of sharding-based blockchain technology and a comparison between those studies, respectively.

### 3.2. Benefits of Sharding-Based Blockchain Technology Network in Environment Monitoring

This section focuses on determining the significant benefits of sharding-based blockchain network technology in securing IoT sensors. Some benefits include increased security in data storage, transmission, reliability, and transparency, which offer an increased opportunity to monitor environmental emissions and manage the risks causing issues. Moreover, as it is a decentralized platform, it incentivizes sustainable practices by rewarding industries that focus on reducing carbon footprint.

Further, the key findings that determine the benefits of sharding-based blockchain technology are mentioned. As per the findings of [50], increased traceability, scalability, and efficiency are identified. It also results in minimized costs of production activities, which aids in developing more authentic and reliable smart manufacturing systems. Similarly, the findings of [51] also determined several benefits, like the secure transfer of data, better integration of services, monitoring of the IoT, data management, and privacy concerns, which increase the awareness of continuum care. This is also reflected in the findings of [52]. It also saves time by increasing its speed. This significantly helps increase and improve performance for the significant issues of blockchain scalability in developing a scalable electronic voting system. However, ref. [53] propounded that scalability without any limit is considered a potential benefit, as a limited ability to scale leads to restrictions on blockchain technology by limiting its high latency and low throughput. However, sharding is the solution to this significant scalability issue within this blockchain network technology, involving the sharding-based PoW and sharding-based PoS blockchain protocols that help in improving performance to secure IoT sensors.

Further, ref. [34] explained that this technology also provides context-aware sensors that enable the gathering of information from the user-patient environment to support medical provision treatment. Ref. [54] determined the cost-effectiveness of securing IoT sensors. Moreover, it also results in showing the significant solutions by the electronic voting systems based on the blockchain technology that help in attempting to determine the future developments by selecting the significant proposals, implementation methods, and cryptographic solutions, along with verification methods. As determined by the study of [55], off-chain and on-chain techniques further result in optimizing resource use, maintaining data integrity, and enhancing scalability. These technique themes are further helpful in addressing the significant issues of blockchain bloat as the on-chain technique updates the consensus mechanism, database partitioning, and block size, and the off-chain technique processes parallel, handling transactions and bundling transactions. The findings of [55] indicated that a tree-assisted inter-sharding consensus helps in safeguarding against Sybil attacks, which helps in the efficiency and security of the technology. Similarly, the findings of [32] explained an increased decentralization to process and generate large volumes of data. Further, the issues of network technologies and edge computing can be effectively resolved by utilizing new edge computing architectures and edge nodes, along with compression methods that effectively demand embedding the deep neural networks within the edge devices. As opined by [56], increased data dependability and security in the IoT systems involves data blocks in blockchain technology, with complete security to assess the consistency of all data. This can be understood by designing a problem methodology that is clustered with a load balancing technique, and is integrated with the data weights by ensuring that the parametric evaluations are performed in real-time by determining the consistency of every data item that is monitored using the IoT. For this, ref. [53] demonstrated sharding-based technology to be highly effective. Another study by [57] shows interconnecting with objects and energy-efficient offloading that further aids in managing traffic in the IoV systems, leaving a reliable impact on the environment. However, ref. [34] determined that the integration of the IoMT has also resulted in increased risks of security issues, which further result in foreseeing the blockchain network technology as a disruptive technology that later on integrates into an effective solution for the integration of IoMT networks, which play an important role in securing these devices, and also help in abstaining from unauthorized access at the time of data transmission. Moreover, a virtual machine environment is effective through the implementation of an authentication, registration and consensus algorithm to compare the BlockAuth scheme with other related schemes within the healthcare environment. This scheme further provides several benefits in big data tolerance, strong reliability, security, and multi-signature identity data. However, the findings of [58] explained that the integration of the IoT has significantly benefited from revealing the lack of openness in the agricultural supply chain, tensions regarding financial losses, reduced corporate brand value, and eroded customer trust, offering a safe, sustainable, and equitable process that meets the customer’s demands. Similarly, ref. [37] explained that it enhances the robustness of the classification responses that were demonstrated during plant diseases.

Similarly, the findings of [59] offered homomorphic encryption, facilitating the calculation performance of the encrypted data, which also safeguards the privacy of data through computational processes, which protects the privacy of patients. Further, the findings of [60] described that implementing sharding-based blockchain technology involves transparency, data integrity, and decentralization. Similarly, ref. [61] determined increased trustworthiness and security of IoT networks, which helps in the collection of sensor nodes and effectively compressing data. It is also a significant element of the blockchain system that effectively classifies and processes data depending on the confidentiality levels by assessing the cloud server storage for increased security.

**Figure 3:**
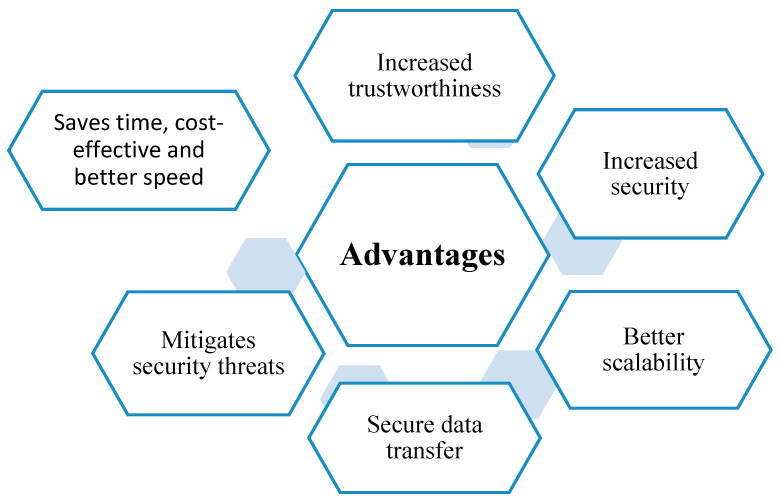
Benefits of sharding-based blockchain technology in securing IoT sensors.

Some benefits of sharding-based blockchain technology are shown in Figure 3.

According to the findings of [44], the significant benefits involve enhanced capacity, security, anonymity, peers’ capabilities, and smart-contract-related features. However, ref. [43] demonstrated that low power consumption is one benefit, and it offers significant benefits such as effective ledger functions, decentralization, encryption, and time stamps, and significantly has the potential to deal with security issues, resulting in cost-effectiveness. Moreover, ref. [62] explained that blockchain offers a practical solution that significantly aids in the improvement of transactions per second. Optimal blockchain sharding complexity is considered a Markov decision process, focusing on the number of shards, block interval, and block size. The study has significantly involved block intervals and size, considered as continuous decision variables that offer significant strategies for dynamic sharding. The results have further determined that the BDQBS helps overcome the high-action space limitations and complexities of training in neural networks. In addition to this, the findings of [63] determined that it helps develop trustworthy and secure decentralized transaction storage through immutable and related transactions, which mainly depend on the significant trustworthiness of knowledge data along with AI model sharing. It also aids in traffic optimization, access control, traffic filtering, the detection of anomalies, data analysis, cloud computing, access control, resource sharing, protecting privacy, data center networking, and network visualization. Similarly, ref. [62] also helped in improving transactions per second by offering a practical solution and addressing limitations and complexities. Further, the results of [48] involve benefits such as the availability, integrity, and confidentiality of protecting data. The results have further determined that, through blockchain network technology, decentralized solutions can be offered to help resolve trust-associated difficulties. Table 4 and Table 5 present a summary of studies on the benefits of sharding-based blockchain technology with their main contributions and a comparison between those studies.

### 3.3. Challenges of Sharding-Based Blockchain Network Technology in Environment Monitoring

Sharding-based blockchain technology is effective in monitoring the information gathered by IoT sensors without permitting them to copy any wrong data. However, in this case, several key challenges that can be determined are identified from the existing literature. Some of the challenges include interoperability, power consumption, scalability, data personalization, storage, transactional costs, and digital rights management.

As propounded by [65], BrokerChain is required to handle security issues. Also, the imbalanced transaction is induced because of the account deployment mechanism. To address these concerns, transaction-driven simulators and cloud-based prototypes help outperform the solutions by balancing their workload, system throughput, and transaction confirmation latency. The study by [66] explores power consumption and scalability as major challenges. Using Bitcoin results in immersion cooling in blockchain mining, with a 50% chance of attacks. Scalability results in increasing the size of the network and its scalability issues, with an increased number of transactions, around 1,668,000 transactions per second, with a 2 Gbps fiber network decreasing. This issue compromises confidentiality, increasing the likelihood of splits in the blockchain topology. With this focus, ref. [58] explains that the network also preserves data privacy. Further, the results of [67] determine that the blockchain IoT is heterogeneous and dynamic in nature, due to which it fails to reconfigure shards. However, with the help of PPO and TPPOD, sharding technologies can focus on shard reconfiguration, especially when the environment changes. In addition, ref. [23] determined scalability issues due to inefficiency in inter-peer communication. This enhances the need to create optimization based on a network offering an optimization framework that analyzes the scalability. Furthermore, ref. [51] determined interoperability, data personalization, storage, and transactional costs to be challenges. These issues affect the legacy care settings and legal issues in the health and care settings, which effectively require enhanced attention to focus upon. Meanwhile, the findings of [68] explain that in order to build a trusted edge computing environment, it is significant to implement blockchain-federated learning. The results indicate that through DAG main chain, a 14% improvement in training efficiency is determined within FL systems. Further, based on the findings of [69], sharding was determined to be the best way through which blockchain performance can be improved. With the help of a replay epoch, more than 50,000 TPS are used under the transaction of consumers to determine real-world shopping behaviors. However, ref. [53] found that security issues, reasonable latency, good throughput, and performance are some of the significant challenges. Similarly to these, refs. [52,66] also determined scalability as a major barrier that works as an obstacle within the electronic voting systems and increased network size with blockchain technology through various data privacy, verifiability, integrity, transparency, and authentication issues. Similarly, ref. [70] also demonstrated scalability and cybersecurity as major problems. However, the sharding-based technology effectively addresses these issues in implementing IoT, with the help of real-time processing of validation trade-offs. Further, it shows reliability issues in securing IoT sensors. Additionally, the findings of [54] explain that the loss of data and privacy and security concerns are challenges. As blockchain bloat shows an increased growth of data on the blockchain networks, it also presents a significant challenge, which mainly arises from less knowledge in the field. Further, the study by [30] explains that securing communication is still a challenge between IoT devices because the sensing devices are resource-constrained and have limited processing capabilities. Similarly, ref. [68] determines several challenges in handling large-scale FL tasks, due to limited transaction throughput, difficult communication requirements, and substantial resource consumption. As determined by [44], there are issues in the consistency of transactions with an increased number of nodes. Through HBFT, the scalability to assign nodes to various layers further results in an improvement in reducing communication difficulties. These communication difficulties are reduced as every node exchanges messages within its group. Further, the findings of [29] determined the data security needs of IoV by meeting the existing nodes and significantly undertaking the computation process and caching. This results in alleviating the storage pressure within the sharding-based blockchain nodes, which affects the blockchain performance because of frequent cross-shard communication. Similarly, ref. [49] determined that the several challenges include privacy and security concerns. It also results in maintaining encryption, decoding speed, and increased transmission. The findings of [71] have explored that traceability issues also exist within the blockchain network technology, leading to increased privacy risks within the organization. On the other hand, the author has revealed that there are several benefits, and significant mechanisms, such as the basis of confidential computing, trusted execution environment, and secure multi-party computation, help deal with the significant issues of data privacy within blockchain technology.

As propounded by [72], the problems of scalability and reduced performance result in increased risks in the trust management in blockchain technology because of privacy and security risks; however, sharding-based blockchain networks help in addressing these issues of scalability and accommodate larger access to devices. Further, the efficiency of transaction processing within the blockchain-based trust management solution results in increased scalability. The findings of [59] determined that increased risks of confidentiality and protection are the significant challenges of integrating IoT within the shared blockchain network. Additionally, ref. [39] explained the issues of cybersecurity, requiring different techniques to overcome these issues. Further, the results of [38], explained with the help of its benefits, resolve its issues of scalability. The study findings have also determined the technique that results in eliminating cross-shard communication by completely forming the shards. Similarly to the findings of [59], ref. [73] determined that the implementation of the IoT focuses on developing reliable and robust applications. The study’s findings further determine the need for decentralized marketplaces for IoT data that later exploit blockchain technology by providing significant benefits, matching the effective needs of the marketplace, and helping make insightful decisions for the new market. However, ref. [59] has also determined several significant challenges. In addition, the findings determined by [39] explain the issues of cybersecurity in blockchain technology in IoT systems. Issues such as resource constraints and privacy concerns also pose challenges that require privacy-preserving techniques, lightweight cryptography, and efficient consensus mechanisms that help deal with these issues. Similarly, the study by [59] also determines cybersecurity challenges, which require reliable and secure deployment. The study’s findings have focused on discussing the significant issues of cybersecurity by understanding the different areas, which are IoT security devices, blockchain networks, and the implementation of IoT devices, along with blockchain. Moreover, as determined by [42], challenges include scalability issues/storage capacity issues, the scalability of transaction rates, resource utilization, legal issues, and predictability. However, the results demonstrated by [41] found challenges such as scaling, interoperability, and security concerns, as the technology involves heterogeneous and dynamic computing, along with communication requirements. It also involves security concerns, which must be considered during IoT and BCN operations and deployment. Further, this has been determined by [74], who explained complexities like reliability, trustworthiness, confidentiality, and security. Security is a significant aspect of cryptography, which focuses on patient health records. However, ref. [75] determined that IoT devices have been effective in accelerating the transmission process of data, which further caters to the increased demands of IoT users. This determines that data transmission based on IoT mobility is one of the most challenging issues. To address the security concerns of blockchain network technology, a significant decentralized and branched blockchain network has been effectively formulated, which helps facilitate the side-channel attacks of the IoT. Further, the results of [63] determined several issues, like the difficulty in processing large data, the increased consumption of energy, and scalability problems. Further, the findings also focus on identifying significant solutions to address the challenges by considering dissemination and knowledge generation. Moreover, ref. [76] demonstrated issues in its performance, scalability, complexity, and cost. The study’s findings have significantly focused on addressing the complexities of blockchain network technologies relating to performance issues, scalability, mobile-specific constraints, and data storage costs, while the results of [77] determined that extended transaction confirmation times are a significant challenge. This challenge is caused due to the isolated states between the allocated strategies of shards and unbalanced transactions. Because of this, there is a significant increase in the disproportionate shard workloads and cross-shard transactions which results in delaying the cross-shard transactions. These can be mitigated with the help of the PFQN model [77] and significantly incorporating a novel confirmation queue that effectively focuses on accurately simulating the actual transaction confirmation process in the blockchain network. In addition to this, ref. [78] determined that the increased usage of electronics has resulted in increased e-waste that leads to an increased usage of natural resources and generates hazardous substances. However, the EEE value chain can be adopted to reduce the issue of e-waste within the environment and contribute to a sustainable value chain. However, ref. [78] indicated that the blockchain implementation system shows a defect in insufficient sustainability. However, to address the issues of blockchain, sharding-based technology is effective in maximizing the transaction process by splitting the blockchain into several blocks.

As IoT nodes are resource-constrained devices, it effectively assesses the effect of sharding-based blockchain solutions on energy consumption. It determines the dynamic algorithm, energy-efficient consensus, data compression, adjusting block size, and interval, and the implementation of energy-efficient data can further be explored for large-scale deployment involving the clustering of nodes, edge computing, green networking, and fog computing [63]. On the other hand, ref. [44] explained that different consensus mechanisms, including PoS, PoA, and PoC, are the most energy-efficient mechanisms compared to the PoW mechanism. With the help of these mechanisms, an increased energy trading framework on blockchain can be performed for secured energy transactions through edge computing between smart devices to serve customers and suppliers.

Contrary to this, traditional consensus mechanisms are not considered effective for IoT devices, mainly because of their resource-constrained nature. Hence, the development of energy-efficient and lightweight consensus mechanisms helps to address these issues in IoT devices while ensuring the integrity and security of the blockchain network through fog computing [59]. Table 6 and Table 7 present a summary of studies on the challenges of sharding-based blockchain technology with their main contributions and a comparison between them, respectively.

### 3.4. Security Issues of IoT Sensors in Various Aspects

- Firmware updates: These are special software that are directly placed into device hardware which can be easily installed and uninstalled from the IoT device. However, they also have security issues due to weak authentication, insider threats, a lack of encryption, tampering, alteration, storing sensitive information, and a lack of update mechanisms. As compared to sharding-based technology, these lead to unauthorized access, data tampering, the denial of services, and MitM attacks [22].
- Data routing: These security issues affect the dynamic environment which is prone to routing attacks. These can occur due to various reasons, like physical vulnerabilities, a lack of physical hardening, inadequate update mechanisms and password protection, poor device management, and MitM attacks. In comparison with sharding-based blockchain technology, it results in eavesdropping, routing attacks, MitM attacks, DoS, and data tampering [24].
- Encryption: Encryption is another important threat that should be focused on IoT devices. These result in corrupting the data by compromising the security of IoT devices, along with weak passwords that can be easily cracked by attackers, and poor encryption which put the data sending and receiving information at risk. Compared to sharding-based blockchain technology, significant encryption issues such as data integrity, confidentiality, authenticity, scalability, and key management can occur [46].
- Communication: Security issues relating to communication also occur within IoT devices due to DNS threats, leading to issues including data integrity, firmware exploits that can be exploited by attackers, insecure interfaces leading to security issues, and brute force attacks where attackers use possible username and passwords to find the correct combination. Compared to sharding-based blockchain technology, several communication security issues such as replay attacks, MitM attacks, DoS, eavesdropping, and data tampering can occur [29,78].
- Resistance to cyberattacks: Mostly, IoT devices largely focus on connectivity instead of security, which leads to the inadequate management of the device because of a lack of built-in safeguards, not supporting the installation of endpoint agents, not producing logs, and an inability to be updated and patched easily. In comparison with sharding-based technology, it can result in Sybil attacks, eclipse attacks, DoS, DDoS, and quantum computer attacks [43,54].

### 3.5. Existing Projects That Have Implemented Blockchain Technology in the IoT

Blockchain technology in the IoT has significantly impacted the industry, further enabling the industry to connect to billions of devices over the internet and generate unprecedented amounts of data [2]. This technology has been highly involved and integrated within various fields, from smart cities to vehicles and wearables. Hence, dependency on the IoT blockchain has been increasing. With this focus, there is an increasing concern regarding privacy and security issues with implementing blockchain technology in different real-world projects [7]. It further plays a crucial role in shaping the digital future and opening new opportunities and possibilities for different businesses and industries.

In the real world, smart cities have effectively implemented the IoT and blockchain technology, and work together to enhance people’s daily lives. The integration of this technology relies mainly on large networks of interconnected devices, which is effective in traffic and flow management [2]. However, it also creates a large amount of data which must be handled efficiently and securely. With the help of these advanced technologies, transparent and secure data sharing between stakeholders and city departments can be enabled, effectively managing resources like water, energy, and waste. Further, it can also enable new business models by permitting individuals to monetize their information and maintain control over privacy concerns.

Agriculture is another field that largely uses the IoT blockchain and offers various advantages such as traceability, certifications, and monitoring and management of the crop [57]. By integrating this technology, end-to-end traceability of products can be achieved while ensuring the safety of food products and compliance with the rules. It also effectively certifies fair trade, organic, and sustainable agricultural practices. Further, it helps gather data and information on soil moisture, crop health, etc. These data later help optimize crop management, such as pest control, irrigation, and fertilization.

The IoT blockchain is also a game-changer in the medical field, facilitating electronic health records and medical device tracking. EHRs help collect and secure patient records and store them in the decentralized ledger while giving authorized individuals access [34]. Furthermore, medical device tracking effectively helps trace the product’s journey by preventing it from being forged and entering the market.

Furthermore, Harmony is another blockchain project that focuses entirely on sharding-based networks for scalability and emphasizes achieving higher throughput while managing decentralization. It primarily focuses on state sharding, fast consensus, and cross-linking. It shards the account balances and smart contract storage instead of fair transactions. This effectively helps in avoiding a communication overhead between shards. Harmony also uses FBFT, which involves a set of validators that effectively help optimize cross-shared communication. It effectively ensures cross-linking between the shards to maintain consistency so validators can actively participate in their cross-shard and shard consensus.

Zilliqa is another significant sharding blockchain project which implements sharding to achieve high throughput without compromising security. It involves network shards, divides its network into various shards, and can process transactions parallelly. Each shard has its consensus, which helps gather and validate transactions. It uses a smart contract language called Scilla, which is sharded and helps execute concurrently across various other shards. Lastly, dynamic sharding is effective in adjusting the number of shards depending on the demands of shards. The increased or decreased number of shards can result in the addition or removal of shards in industries.

## 4. Discussion

This discusses the findings from the results of the literature review based on the research questions. It is effectively helpful in providing a sound understanding of securing IoT sensors through the integration of sharding-based blockchain network technology.

### 4.1. Impact of Sharding-Based Blockchain Network Technology in Securing IoT Sensors

From the determined results of the literature review, it can be discussed that sharding-based blockchain technology is one of the types of blockchain technology that is considered effective in database partitioning techniques. This effective technique is utilized to enable scalability within blockchain network technologies, which is further helpful in processing more transactions per second. Through comparative analysis, the findings have significantly determined that the use of sharding-based blockchain technology shows a positive effect on securing IoT sensors by offering feasible solutions for navigating the challenges of IoT sensors through improved efficiency and adap and versatile simulators [25,26]. The use of a permissioned blockchain solution through Hyperledger Fabric helps ions, reducing the scalability attrition which effectively helps to deploy blockchain on a larger scale through practical and extensive experimentation, indicating the impact of service quality within blockchain networks to demonstrate feasible solutions [21]. The stability and performance of sharding-based blockchain technology are not compromised, and the reliability and efficacy of the system help the user understand if the system is securely developed and if there are any scalability updates.

**Figure 4:**
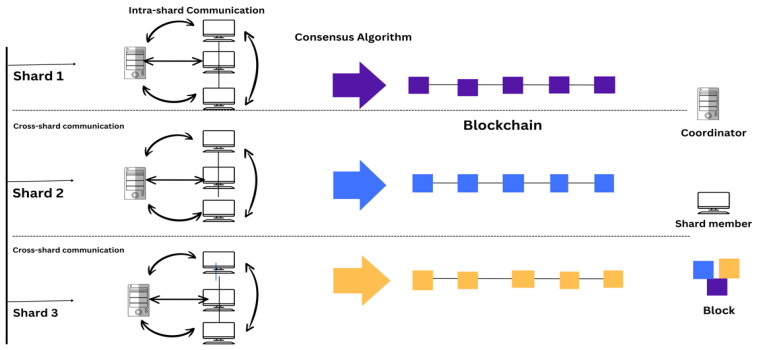
Communication in sharding-based blockchain technology, where nodes in the same shard has to perform intra-shard communication and provide key information to coordinator. This is responsible for cross-shard communication, and intra-shard communication among the blocks in purple, blue, and yellow color.

Moreover, inefficiencies in peer communication have also been identified as providing an important effect on the integration of the sharding-based blockchain network, which can be addressed through flooding techniques by effectively sharing blocks and by producing unchangeable logs of transactions, effectively communicating with IoT nodes by conducting a survey [23]. On the contrary, through a systematic review, the results have determined that storage efficiency is one of the concerns for IoT sensors within the blockchain network technology, and improved storage efficiency is offered as a better solution for the blockchain technology that helps address the issues of storage inefficiency, providing valuable insights for long-term sustainability [33]. Another study determined the positive integration of sharding-based blockchain technology with the help of simulation analysis, and has also resulted in determining that communication is effectively possible with the help of blockchain technology by ensuring the better security, confidentiality, availability, and integrity of IoT sensors [24], as shown in Figure 4. It also indicated a minimum encryption time of 2107 ms and file size of 1531 kb. This shows that the shards communicate through cross-shard communication using the consensus algorithm.

By applying the Ethereum blockchain experiment with 50 client nodes for federated learning, sharding-based technology has been considered effective as it helps in the aggregation of a learning process, providing a secure update to the system by determining the significant bottlenecks [27]. The results also determine that sharding-based blockchain network technology has been effective in the improvement of intra-shard transactions that enhance the security of the blockchain network technology, while securing IoT sensors with the help of a motivation mechanism through theoretical analysis and conducting experiments [38]. In addition, Anchorhash has also been helpful in reducing node mapping, enabling blockchain technology to be more effective, scalable, secure, and decentralized [28]. This effectively indicated the improved process of cross-shard transactions, offering better security for the system along with shard efficacy.

The findings have also demonstrated that IoTs’ connection with the internet is effectively achieved using sharding-based blockchain technology, resulting in higher productivity by determining countermeasures against cyberattacks with the utilization of the Oracle model. This aids in improving the security of the system, resulting in determining the significant impact on IoT performance through the determination of randomness and unpredictability [29]. Sharding-based technology is also helpful in affecting the overall performance of the system, which significantly results in optimizing the nodes, focusing on enhancing the cache-hitting ratio and reducing delays within the blockchain network system, further leading to enhanced performance through the content- and node-sharding method [39]. This technology has been considered quite effective in the exchange of data, as it produces unchangeable transaction logs and also communicates with various IoT nodes by involving real-time analytics, embedded systems, and commodity sensors to create immutable records of the transaction history; however, vulnerabilities and reliability in securing the IoT affect blockchain performance [40]. These findings have been determined using the export consensus method, the Schnorr signature method, and the random-based lightweight algorithm. By proposing a blockchain-enabled Internet of Things architecture framework, ref. [33] produced unchangeable logs of transactions, further discussing difficulties including issues of storage, scalability, privacy, and interoperability; applications; and trends between the IoT nodes of the framework.

By conducting a systematic study, it has been determined that the integration of sharding-based blockchain network technology helps provide significant solutions to address privacy and security issues within the IoT, as blockchain nodes have vulnerabilities that affect the performance of the IoT. The chain layer incorporated within the multi-level blockchain technology is quite helpful in ensuring the reliability of the data, and is also effective in lightening the weight. Furthermore, it also results in maintaining the ledger’s copy and validating the transaction process, along with the implementation of IoT sensors [41]. The significant issue of integrating blockchain technology with IoT sensor nodes is difficult. However, the scalability feature of sharding-based blockchain technology helps address this issue, which has been demonstrated by conducting a systematic review [45]. Contrarily, significant advantages involving peer-to-peer capabilities, anonymity, enhanced security, and capacity have also been determined; however, akin to the above study, significant challenges, including resource usage, storage capacity, legal issues, predictability, and transaction rate scalability, have been determined by conducting a systematic literature [42]. Also, the increased demand for data scaling has resulted in the secured data of IoT sensors. The significant knowledge of data-sharing policies within sharding-based technology makes use of encrypted data to effectively generate an audit of transactions that helps understand the accountability and transparency of the system with the help of resource optimization approach [47]. On the contrary, by conducting SLR, sharding-based blockchain technology effectively offers advantages, as well as disadvantages, in securing IoT sensors. The several limitations include the limited transaction performance and limited privacy protection [48].

### 4.2. Benefits of Sharding-Based Blockchain Technology Network in Securing IoT Sensors

The results of the literature review have effectively determined that the integration of sharding-based blockchain technology has been advantageous in securing IoT sensors. Some of the significant advantages of integrating sharding-based blockchain network technology include the secure transfer of data, the better integration of services, and the awareness of continuum care by conducting a survey. Scalability is the most significant benefit of integrating sharding-based blockchain technology, by offering PoW and PoS protocols that result in better performance in securing IoT sensors through a probabilistic model. Furthermore, off-chain and on-chain techniques are also helpful in the optimization of resources, better scalability, and maintenance of data integrity that leads to handling and bundling transactions by exploring practical insights [53,60]. Similarly, ref. [54] determined scalability as a benefit based on proposed on-chain and off-chain techniques, along with significant limitations and recommendations including resource usage optimization, scalability, and data integrity. Additionally, the increased traceability, transparency, and security offered better results within the system, which has been determined by conducting a survey for smart manufacturing. Further, several limitations such as system integration, scalability, and privacy protection also exist [50]. Another study [51] demonstrated the advantages and integration of blockchain with ambient assisted living with the help of systematic reviews, and PRISMA. Another benefit of sharding-based blockchain technology is that it is a secure and effective technology that helps in preventing targeted attacks based on the node state. The utilization of data dependability and security results in consistent data after the integration of this sharding-based technology through an offloading technique. These consistent data are effective in processing accurate data in the case of IoT sensors. In addition, to address the concerns of privacy and security issues within this system, transparency, data integrity, and decentralization, along with better capability and anonymity, are effective which is determined through parametric evaluations [56]. Another study determined the combination of shard recovery and its reconfiguration, which effectively helped prevent Sybil attacks by conducting a performance evaluation test [55].

The use of dynamic sharding-based technology helps enhance the transactions of every second which can effectively result in addressing the training issues. These technologies are effectively beneficial in safeguarding privacy, network visualization, detection of anomalies, traffic filtering, traffic optimization, resource sharing, administration, access control, and data center networking. The results from the simulation test indicate that the system also involves a distributed and immutable architecture that focuses on resolving the difficulties of partitioning among various shards within the blockchain nodes, by integrating an effective solution for implementing the IoMT networks to enable gathering information from the user–patient environment in supporting medical provision treatment with the high throughput factor k, where k determines number of shards [34]. Also, with an increase in nodes from 50 to 100, 150, and 200, it also leads to increased consensus latency. Ref. [38] proposed a transaction-based sharding technique and used simulation experiments indicating the outperformance of standard healthcare blockchain techniques considering the throughput, appointments processed, and consensus latency.

By conducting a systematic literature review, the results indicate that sharding-based technology has increased efficiency and traceability, which results in decreased production costs. It also provides a significant solution, offering a cost-effective method of implementation. This leads to saving time, as the speed is higher by increasing result processing, decreasing and controlling the chances of fraud, and minimizing human involvement; also, blockchain offers a safe, sustainable, and equitable process with decreased financial losses, and increased brand value and customer support and trust to meet their needs and demands [58]. Contrarily, ref. [52] conducted SLR and determined effective solutions related to data privacy, and integrity, authentication, verifiability, and transparency in electronic voting, along with limitations leading to the inapplicability of digital interactions. However, with the help of a multi-phase system, scalability helps assign the nodes, which is effective in resolving communication difficulties. Furthermore, the BDQBS algorithm results in a trusted execution environment, multi-party computing, and secure multi-party computation are also some of the significant advantages of the blockchain system. Along with this, the security of IoT networks and trustworthiness gather sensor nodes and result in system integrity and authenticity by involving block intervals, and size for dynamic sharding further, utilizing BDQBS to address the complexities and limitations of training, such as high-action space issues [61,62]. Similarly, the findings determined by conducting a systematic review indicated that reduced power consumption within the system aids better ledger functions, time stamps, decentralization, and encryption to address the problems of security issues, and is cost-effective, but includes limitations such as limited scalability and limited privacy protection [48]. The availability and integrity of the system offer decentralized solutions to provide enhanced security through secure decentralized transaction storage depending on the trustworthiness of knowledge data which is determined through survey methodology [63]. The integration of sharding-based blockchain technology helps provide homomorphic encryption by effectively reviewing the performance of calculations to prevent data privacy. Moreover, the system is considered effective in understanding and abstaining from unauthorized data transmission [34]. Lastly, the integration of this technology helps determine any lack of openness in the supply chain management, which can lead to significant issues such as financial losses, eroded customer trust, and lowered corporate brand value with the help of Agri-SCM-BIoT. This integration is considered highly beneficial as it offers a sustainable and equitable process that results in meeting the needs of its customers; indeed, enhanced capacity, peers’ capabilities, smart-contract-related features, anonymity, and low power consumption are effective in offering decentralization, time stamps, and encryption to offer cost-effectiveness to the customers [58]. Contrarily, ref. [43] determined latency, network block loss percentage, anomalous temperature, bandwidth, and BTR to identify DDoS attacks by conducting experiments leading to device hardware failures, showing block loss percentage over 50% and also 81.3% high.

A comparative analysis of sharding-based blockchain technology is also discussed by comparing it with traditional blockchain, sidechains, and DAG. Traditional blockchain does not offer scalability features which limits the transaction process, is energy-intensive, and is also vulnerable to attacks, while the sharding-based technology offers increased scalability and is energy-efficient.

Sidechains are also vulnerable and complex systems which are not scalable, further limiting the transaction process, while sharding-based technology is simple, secure, and scalable, enabling the blockchain to process large transactions. Additionally, DAGs are also vulnerable to attacks and security risks. They offers scalable features, but are complex in nature, requiring large computational transactions, while sharding-based technology enables the blockchain to divide into smaller shards to process large transactions parallelly.

### 4.3. Challenges of Sharding-Based Blockchain Network Technology in Securing IoT Sensors

IoT sensors are used in various applications, especially in data processing systems which further require high security as the data are stored in cloud monitoring systems. Despite the significant benefits of sharding-based blockchain network technology, several limitations also exist. The results of the literature review have determined that power consumption is one of the complexities that relates to computational power, causing issues with immersion cooling. Significantly, scalability is also a major issue within the blockchain system, which is determined through the results of a simulation that results in increased splits along with latency and lightning networks, creating inefficiency in inter-peer communication, with 51% chances of PoW attacks [23,66]. Ledger replication and consensus mechanisms are also the cause of scalability issues within the blockchain system determined through simulation. Trust mechanisms, issues, and data security are significant challenges that result from the smart manufacturing of IoT sensors. This also causes inefficiency in their communication with peers. Ref. [72] also determined scalability as a major limitation, determined through simulation results based on real-world data, explaining that the trust-management model exhibits better scalability compared to existing approaches. Similarly to this, ref. [76] utilized a BSADS framework involving six frameworks and determined scalability as the major limitation, along with mobile specific constraints, data storage costs, and performance, which requires a highly technical optimization technique to address these issues. The results of [52] determined that scalability is a fundamental limitation that will affects electronic voting in the future. By conducting SLR, it further demonstrated effective proposals, implementations, methods, and approaches to address this issue. Similarly, scalability, security, and privacy are found as limitations by [42], who determined this by employing a survey methodology based on architectural and operational perspectives. Additionally, it also has resource utilization and legal issues, deploying smart contract limitations. Moreover, ref. [23] again determined scalability as a limitation, including the complexity of blockchain storage requirements, which can be effectively addressed through inter-peer communication. The random neighbor selection technique is used to exchange the blockchain data while analyzing the efficacy of the system.

**Figure 5:**
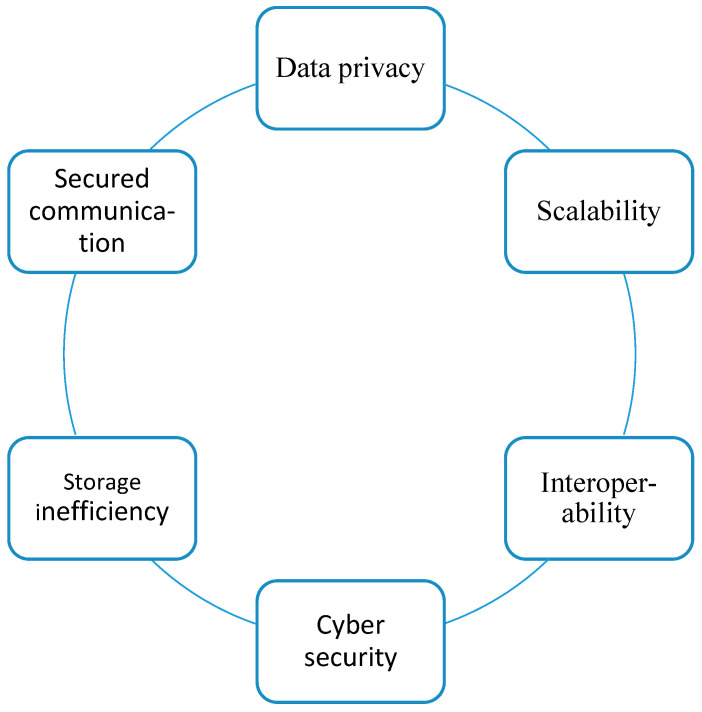
Challenges of sharding-based blockchain technology.

Figure 5 represents a diagram that highlights the main challenges of sharding-based blockchain technology.

Transactional costs, interoperability, data personalization, predictability, scalability of transaction rates, and digital rights management, along with security issues, affect the legal issues and legacy care settings that lead to more issues within the blockchain system by securing IoT sensors through comprehensive evaluation [51,65]. On the contrary, ref. [53] conducted a probabilistic model and determined the security of the sharding-based blockchain technology, indicating a failure of approximately 4000 in a 4000 network of nodes, along with 10 shards, and resiliency of 33%. Some of the limitations include the specific structure of the blockchain protocol, and an insufficient review of the objective. By conducting a survey, it has also been found that energy constraints, along with limited memory and processing, make it difficult to complete the task within the system. Furthermore, storage inefficiency is also another significant concern that requires increased ledger size and storage for the technology; it effectively results in inefficient transaction processing within the blockchain-based trust management, leading to enhanced scalability [25]. The case study determined that cybersecurity issues are also a significant concern, leading to reliability issues, including block propagation, node assignment, and lowered efficiency affect the system negatively, leading to disadvantages; it also results in maintaining encryption, decoding speed, increased transmission, along with privacy issues, and resource constraints [49]. Contrarily, scalability is identified as a limitation through the evaluation of results and by conducting normalized mean square errors, indicating the reputation scheme to be less than 0.2, and TPS performance as 1.4 times promotion of 1000 nodes [65]. Similarly to these, ref. [59] indicated issues such as confidentiality, reliability, security, and trustworthiness, aided by the homomorphic encryption technique. It indicated better security, and a trustworthiness mechanism within the healthcare system. On the contrary, ref. [39] determined limitations such as scalability, immutability, and decentralization as issues affecting blockchain performance in securing IoT networks, with the help of a comprehensive framework. Furthermore, cybersecurity issues are also common issues in the integration of sharding-based blockchain networks, and strongly require secure deployment. A lack of knowledge and understanding, along with its secure communication, is also a major concern for the system. The bad performance of the sharding-based blockchain technology is just because it involves cross-shard. Traceability within the system is an important challenge that can lead to an increased risk of privacy, and securing communication is also a challenge between IoT devices [71]. Akin to the above findings, ref. [30] determined making communications secure as a major limitation, because of its resource-constrained nature and the processing capabilities of sensing devices, by employing the AGSK mechanism and random oracle model, which counteracts against cyberattacks. It indicated minimized CPU computation time by 41.3%, and storage cost by 45.7%, and energy consumption by 40%. The bad performance that results in securing IoT can lead to increased risks of management which makes it a not-so-suitable technology. Data transmission also acts as a challenge for IoT-based blockchain systems that leads to enhanced latency, power consumption, side-channel attacks, and computational overhead. Moreover, the cost of implementing the technology is also a challenge, as it leads to decreased performance, scalability, mobile-specific constraints, and data storage. Along with this, by incorporating the PFQN model, the results determined that delayed transactions due to disproportionate shard workloads can lead to isolated states among the allocated strategies of shard; also, issues in transaction consistency increase with a large number of nodes [77]. In addition to this, uncertain authenticity and limited storage for IoT data within the blockchain system have to face challenges for centralized data. Confidentiality, data privacy, security threats, and protection are also challenging within the blockchain network for securing the IoT. Lastly, the results also explained the findings of [44], demonstrating that the security, efficiency, and scalability of the consensus protocol is challenged by increased nodes in the system. By employing the HBFT mechanism, the scalability of the system was improved by assigning nodes within different layers. It further demonstrated the security of the blockchain through random selection mechanisms, and enhanced performance through the consensus protocol.

Some of the security issues involve:

- Replay attacks.
- Consistency issues.
- Synchronization issues.

Determining these different types of security risks, several possible solutions are also mentioned to address these issues. These are:

- A cross-shard transaction protocol to ensure the security and consistency of transactions.
- Synchronization mechanisms to ensure that all the nodes view the blockchain state in a similar manner.

### 4.4. Gaps

A large number of existing studies are present across the internet, with extensive knowledge regarding sharding-based blockchain network technology and IoT sensors. These studies have determined how the integration of sharding-based blockchain technology helps secure IoT sensors. The present study focuses effectively on securing IoT sensors through the integration of sharding-based blockchain network technology. The integration of blockchain technology uses various techniques that help support devices and secure data within the IoT. Furthermore, the use of sharding-based technology within the blockchain networks helps secure transactions through nodes. These are quite beneficial in different sectors by relying on IoT. The current study effectively focuses on determining the potential impacts, benefits, and challenges of integrating sharding-based blockchain network technology. This has helped the researcher in acquiring extensive knowledge and understanding of the subject matter. However, there is no extensive research that focuses on developing a framework that helps improve security in the IoT industry through sharding-based blockchain network technology. Moreover, there are a few studies that have focused on enhancing computation offloading and implementing energy-optimized clustering that is effective in safeguarding the IoT environment. So, this can be a literature gap. Moreover, various studies have determined the challenges; however, there are no studies that have focused effectively on developing mechanisms that help reduce the potential attacks on the sharding-based technology. Hence, this is another literature gap that can be taken into consideration in further studies.

## 5. Conclusions

A systematic literature review based on research papers that emphasized the terms blockchain technology and the IoT published in the main scientific editorials from 2022 to 2024 was carried out. This focused on determining the three significant research questions, which were, what is the effect of sharding-based blockchain technology in securing IoT sensors? What are the advantages of sharding-based blockchain technology? And what are the disadvantages of integrating the sharding-based blockchain technology? To continue with this study further, significant editorials from MDPI and IEEE Xplore were chosen. A total of 51 papers were used in the study, where 47 existing studies were from MDPI editorials, and 4 were from Google Scholar. The studies were effectively analyzed by determining their publication year and their interest in blockchain technology and the IoT.

Furthermore, the study has determined the significant positive impact of sharding-based blockchain network technology in securing IoT sensors, based on the systematic review and simulation results showing a minimum encryption time of 2107 ms and file size of 1531 kb. The results and discussion have also focused on determining the benefits and limitations of integrating sharding-based blockchain network technology, based on the systematic reviews included in the study. The significant benefits involve decentralization, better security, cost-effectiveness, reduced energy consumption, increased speed, traceability, efficiency, better services, secure data transfer, and scalability. The results also demonstrated the use of summary blocks within different blockchain applications for optimal storage utilization of blockchain technology [25]. The SLR also demonstrated the use of Anchorhash and jump consistency hash function within the blockchain network to ensure the security of IoT devices [28]. Additionally, the integration of blockchain offered several desirable features to monitor IoT devices along with the integration of environmental sensors, emphasizing the privacy and security aspects, along with sharing and managing the data [50]. The drawbacks of the integration of this technology involve its increased power consumption, scalability, data security, transactional costs, interoperability, data personalization, predictability, scalability of transaction rates, side-channel attacks, increased latency, computational overhead, and cybersecurity.

### 5.1. Future Research Directions

For future research, it would be effective to examine the progression of this advanced technology with an in-depth focus on scalability and performance in securing IoT sensors, which is not explained in detail in the study. As blockchain technology is a decentralized model, it allows its peers to work together and build trust to enhance the business network. Moreover, every peer node performs computations and also communicates with its peers to validate the transaction process so that they can arrive at a consensus, and at the same time update the status of the shared ledger. This is also effective in environmental monitoring and dealing with the challenges of data security. Hence, it is significant to explore the factors that affect the performance and scalability of blockchain network technology in securing WSN IoT sensors in the future.

### 5.2. Contributions to the Research

To contribute to the field of securing IoT sensors with the integration of sharding-based blockchain technology, a comprehensive literature review was conducted from 2022 to 2024, keeping in mind the research context, which effectively identifies significant gaps and helps to provide a consolidated understanding and knowledge of the research field. Furthermore, the study has determined the benefits and drawbacks of integrating sharding-based blockchain technology to secure IoT sensors. Some advantages of incorporating this include reduced energy consumption, increased security, cost-efficacy, improved scalability, and decentralization. However, several challenges have also been highlighted, such as cybersecurity and privacy concerns, increased power consumption, and scalability issues. Thus, the research effectively addresses the research question, determining the impacts, benefits, and drawbacks of this emerging technology, thereby contributing to the academic field. This has resulted in proposing a clear framework to conduct the study, which other researchers can use in the future to work in a similar field. Based on the findings and contributions in the study, significant areas for future research are suggested, which encourages exploring potential improvements and the complexities of the integration of this emerging technology in IoT sensors.

## Figures and Tables

**Table 1:** Themes.

Themes
1. Impact of sharding-based blockchain network technology in securing IoT sensors
2. Benefits of sharding-based blockchain technology network in securing IoT sensors
3. Challenges of sharding-based blockchain network technology in securing IoT sensors

**Table 2:** Summary of studies with their main contributions on the impact of sharding-based blockchain technology.

Reference	Methodology	Consensus Algorithm	Key Findings
Freire et al. (2022) [21]	Primary quantitative method	AISs	Blockchain technology positively impacted the quality of service. It further shows effective and feasible solutions.
Shin and Jeon (2024) [22]	Secondary method	SHA-256 hash algorithm	The drawback determines leveraging Merkle tree-based verification to aid in fortifying the firmware update efficacy.
Antwi et al. (2022) [23]	Primary quantitative method	PBFT, RBFT, WBFT	IoT calls are effectively secured by sharding-based blockchain technology, showing positive effects. Inefficient peer communication can be addressed through flooding techniques via shards.
Albakri et al. (2023) [24]	Secondary method	PTA, SCA	IoT interconnects with blockchain networks and shows positive impacts. IoT environment is effective through data routing and encryption, which helps employ a trust model and improves the security of the communication process.
Kuleto et al. (2022) [47]	Primary method	Unspecified	The use of blockchain helps decentralization, provides security and integrity, and offers anonymity in transaction. This is further helpful in promoting an increase in transaction rates in educational institutions in Portugal, Serbia, and Romania.
Akrasi-Mensah et al. (2022) [25]	Secondary method	Compression algorithm, NSGA-C, MA-DDPG, PoR	Potential impacts show storage efficiency within blockchain technology.
Khan, Hartog, and Hu (2024) [26]	Primary quantitative method	RSA, TSA	The results demonstrated adaptable and versatile simulators showing positive impacts. Efficiencies and intricacies have positive impacts on assessing the convergence of nodes.
Lee and Kim (2022) [27]	Secondary method	PoW, DRL	This shows potential performance bottlenecks and prevents model-poisoning attacks.
Chen et al. (2022) [28]	Secondary method	Jump consistent hash algorithm	Sharding-based technology has helped improve intra-shard transactions, resulting in enhanced security. Signature Anchorhash reduces the mapping of node assignment, which improves the procedure of cross-shard transactions.
Ali and Ahmed (2023) [30]	Secondary method	AGSK	High productivity and countermeasures against cyberattacks offer positive effects. With the help of AGSK, along with a digital signature and hash function, the combiner is integrated within the IoT devices.
Wang and Guan (2022) [44]	Secondary qualitative method	HBFT	Sharding-based blockchain involve issues in the consistency of transactions with an increased number of nodes. The security of the technology is determined by its randomness and unpredictability, which determines its better performance.
Cholevas et al. (2024) [31]	Primary quantitative method	Avant-garde algorithm	It shows unsupervised learning and unsupervised learning-based anomaly detection, showing that integrity and security are an issue.
Prieto and Barroso (2024) [32]	Secondary method	Unspecified	It shows increased decentralization to process and generate large data volumes. It shows efficient blockchain technology with latent and increased scalability.
Zhao and Ding (2024) [29]	Secondary method	TDGA	Sharding-based technology addresses the data security needs of IoV, which affects the blockchain performance. With the help of the content sharding method, caching can be optimized effectively.
George and Al-Ansari (2023) [36]	Secondary method	Hash algorithm	The integration of blockchain plays an important role in determining the technological infrastructure of food security. The food supply chain experienced several untraceable and inefficient corruptions, which led to the blockchain integration.
Marco-Detchart et al. (2023) [37]	Secondary method	Data fusion algorithm	Findings demonstrate blockchain improves sustainable development in the agriculture sector. It shows the real-time data information to the software and hardware components to determine the diseases in plants and improve its robustness and the classification process.
Alam (2022) [33]	Secondary method	Sha-256 cryptographic algorithm	This shows data security and exchange by producing unchangeable logs of transactions and communicating with other IoT nodes.
Ahakonye, Nwakanma, and Kim (2024) [39]	Secondary method	DDoS, prompting control algorithm	The results show increased performance, and are accessible, transparent, decentralized, immutable, and scalable. It has been determined that IDS development is quite accessible, transparent, decentralized, immutable, and scalable.
Na and Park, (2022) [40]	Secondary method	PoW, PBFT	The role of IoT management in blockchain nodes is maintained through edge nodes, which results in vulnerabilities. Through a consensus algorithm and a multi-level structure, the vulnerability can be effectively resolved.
Zubaydi, Varga, and Molnár (2023) [42]	Primary quantitative method	PoA	The IoT has led to significant concerns about privacy and data security. It also offers the IoT significant effective solutions to deal with these issues.
Arachchige, Branch, and But (2024) [43]	Secondary method	DDoS, XXHASH algorithm	It was determined to be vulnerable to attacks of DDoS, which might result in failures in hardware devices with an increased temperature.
Adhikari and Ramkumar (2023) [41]	Secondary method	BCN	It plays a significant role in each node by maintaining a copy of its ledger and validating transactions by integrating IoT sensors.
Taherdoost (2023) [45]	Secondary method	Unspecified	It determined that the scalability feature makes the implementation process easy.
Schmid, Schaffhäuser, and Kashef (2023) [49]	Secondary method	PoW	It results in maintaining security, trustless authentication, and resilience. These help in resolving the maintenance constraints, authentication problems, and security issues of IoT devices.
Zhao et al. (2022) [46]	Secondary method	Hybrid query algorithm	The results determine the space the nodes consume without changing the actual data query state. It also determined that, through hybrid query management, effective querying of transaction data can be performed.
Wenhua et al. (2023) [48]	Secondary method	PoW	Characteristics like a security focus on decentralization, consensus, and cryptography play a significant role. It also ensures its transaction process.

**Table 3:** Comparison between the studies on the impact of sharding-based blockchain technology.

References	Service Quality	Efficacy	Scalability	Security
Freire et al. (2022) [21]	Positively affects the service quality	Effective and feasible solution	-	-
Shin and Jeon (2024) [22]	-	Fortifies efficacy of firmware update	-	-
Antwi et al. (2022) [23]	-	-	-	IoT calls are secured
Albakri et al. (2023) [24]	-	Effective through data routing	-	Improves communication process
Kuleto et al. (2022) [47]	-	-	-	Provides security and offers anonymity
Akrasi-Mensah et al. (2022) [25]	-	Storage efficiency	-	-
Khan, Hartog, and Hu (2024) [26]	-	Effective in node convergence	-	-
Lee and Kim (2022) [27]	Potential performance bottlenecks	-	-	Provides security to model-poisoning attacks
Chen et al. (2022) [28]	Improved cross-shard transaction	-	-	Enhanced security
Ali and Ahmed (2023) [30]	High productivity	-	-	Countermeasures against cyberattacks
Wang and Guan (2022) [44]	Better performance	-	-	Improved security and integrity
Cholevas et al. (2024) [31]	-	-	-	Improved security and integrity
Prieto and Barroso (2024) [32]	Generates large data volume	Efficient technology	Increased scalability	-
Zhao and Ding (2024) [29]	-	Optimizes effectively	-	Enhanced data security needs
George and Al-Ansari (2023) [36]	-	-	-	Vital in food security
Marco-Detchart et al. (2023) [37]	Better robustness and classification process	Effective in determining diseases	-	-
Alam (2022) [33]	-	-	-	Increased data security
Ahakonye, Nwakanma, and Kim (2024) [39]	Enhanced performance	-	Increased scalability	-
Na and Park (2022) [40]	-	Vulnerabilities are resolved effectively	-	-
Zubaydi, Varga, and Molnár (2023) [42]	-	Provides effective solution	-	Concerned about data security
Arachchige, Branch, and But (2024) [43]	-	-	-	Determines vulnerable attacks
Adhikari and Ramkumar, (2023) [41]	Validate transactions and maintain ledger	-	-	-
Taherdoost, (2023) [45]	-	-	Better scalability	-
Schmid, Schaffhäuser, and Kashef (2023) [49]	-	-	-	Maintain security
Zhao et al. (2022) [46]	-	Effective transaction query	-	-
Wenhua et al. (2023) [48]	Better transaction process	-	-	Better security

**Table 4:** Summary of studies on the benefits of sharding-based blockchain technology with their main contributions.

Reference	Methodology	Consensus Algorithm	Key Findings
Guo, Zhang, and Zhang (2022) [50]	Quantitative method	PoW, PoS, DPoS, PBFT	Increased traceability, scalability, and efficiency are several advantages of sharding-based blockchain technology. It helps develop more authentic and reliable smart manufacturing systems.
Florea, Anghel, and Cioara (2022) [51]	Secondary method	IPFS	Several benefits include the secure transfer of data and better integration of services. It increases the awareness of continuum care.
Hafid, Hafid, and Makrakis (2023) [53]	Secondary method	PoS	Scalability is one of the significant benefits if it is not limited. Sharding is the solution to scalability issues through PoW and PoS protocols that enhance performance to secure IoT sensors.
Jafar et al. (2022) [52]	Secondary method	DSA, RSA, ECC	The integration of sharding-based technology results in cost-effectiveness and saving time with increased speed. It shows solutions for electronic voting systems that help determine future developments.
Alzoubi and Mishra (2024) [54]	Secondary method	Unspecified	Consensus mechanism, database partitioning, block size, processes parallel, handling transactions, and bundling transactions.
Lai et al. (2023) [55]	Secondary method	Unspecified	Benefits indicate that blockchain safeguards Sybil attacks, which helps in the efficiency and security of technology. Shard combination recovery and its reconfiguration are also helpful in avoiding targeted attacks, as it is based on a node state.
Prieto and Barroso (2024) [32]	Secondary method	Unspecified	It shows blockchain with increased scalability by meeting the complexities of flexibility, availability, scalability, and bandwidth.
Shitharth et al. (2023) [56]	Secondary method	Unspecified	It shows secure and determine that there is an increase in the data processing accuracy for every IoT application. It utilized a load balancing procedure, data weights, and clusters which determine consistency, further monitored with the IoT.
Vladyko et al. (2022) [57]	Secondary method	PBFT	This interconnects with objects and energy-efficient offloading that manages traffic in IoV systems, leaving a reliable impact. It builds a correct, reliable, and uncompromised data network architecture, leading to enhanced safety for road users with mobile edge computing.
Alajlan, Alhumam, and Frikha (2023) [60]	Secondary method	Unspecified	Significant benefits involve transparency, data integrity, and decentralization. It also offers a significant solution to improve the security and privacy issues of IoT devices.
Bobde et al. (2024) [61]	Secondary method	PoA	It determined the increased trustworthiness and security of the IoT, collecting sensor nodes and effectively compressing data. It also helps ensure the authenticity and integrity of the system.
Zubaydi, Varga, and Molnár (2023) [42]	Primary quantitative method	PoA	The benefits include enhanced capacity, security, anonymity, peers’ capabilities, and smart-contract-related features.
Arachchige, Branch, and But (2024) [43]	Secondary method	DDoS, XXHASH algorithm	It offers benefits like effective ledger functions, decentralization, encryption, and time stamp, and is cost-effective. It revealed significant benefits like transparency, efficiency, and security, offering the best services and mechanisms for the healthcare field.
Ali et al. (2023) [64]	Secondary methods	BSS	The results determined integrating blockchain technology provides cost-effectiveness, transparency, efficiency, and security. It safeguards the privacy of data through computational processes, which protects the privacy of patients.
Liu et al. (2023) [62]	Secondary method	BDQ	Sharding-based technology divides the network into various disjoint groups to process transactions within the systems. It determines that the BDQBS helps overcome the high action space limitations and complexities of training in neural networks.
Wijesekara and Gunawardena (2023) [63]	Secondary method	SIDH, DAG, digital	Blockchain helps develop trustworthy and secure decentralized transaction storage through immutable related transactions. It also aids in traffic optimization, access control, traffic filtering, detection of anomalies, data analysis, cloud computing, and access control.
Wenhua et al. (2023) [48]	Secondary method	PoW	The benefits include the availability, integrity, and confidentiality of protecting data. The results also determined that decentralized solutions can be offered to help resolve trustless difficulties.
Bhat et al. (2022) [58]	Secondary method	PoW, PoS	Blockchain integration has significantly benefited the IoT by mitigating security threats. It results in reduced corporate brand value and eroded customer trust, offering a safe, sustainable, and equitable process.
Pelekoudas-Oikonomou et al. (2022) [34]	secondary method	PoW	Increased integration of the IoMT has played a significant role in offering its citizens a better quality of life. It helps in abstaining from unauthorized access at the time of data transmission.

**Table 5:** Comparison between the studies on the benefits of sharding-based blockchain technology.

References	Scalability	Security	Effectiveness	Trustworthiness
Guo, Zhang, and Zhang (2022) [50]	Increased scalability	-	Increased efficiency	-
Florea, Anghel, and Cioara (2022) [51]	-	Secure data transfer	-	-
Hafid, Hafid, and Makrakis (2023) [53]	Better scalability if not limited	-	-	-
Jafar et al. (2022) [52]	-	-	Saves time, cost-effective, and better speed	-
Alzoubi and Mishra (2024) [54]	-	-	-	Handles and bundles transaction
Lai et al. (2023) [55]	-	Secures technology	Efficiently prevents Sybil attacks	-
Prieto and Barroso, (2024) [32]	Increased scalability	-	-	-
Shitharth et al. (2023) [56]	-	Secures data processing	-	-
Vladyko et al. (2022) [57]	-	-	Energy-efficient and manages traffic	-
Alajlan, Alhumam, and Frikha (2023) [60]	Increased transparency	Improved security	-	-
Bobde et al. (2024) [61]	-	Increased security	-	Increased trustworthiness
Zubaydi, Varga, and Molnár (2023) [42]	-	Better security and anonymity	-	-
Arachchige, Branch, and But (2024) [43]	-	Improved security	Effective decentralization and encryption	-
Ali et al. (2023) [64]	Provides transparency	Offers security	Cost-effective and efficient	-
Liu et al. (2023) [62]	-	-	Effectively overcomes space issues	-
Wijesekara and Gunawardena (2023) [63]	-	Secured transactions	-	Develops trustworthiness
Wenhua et al. (2023) [48]	Offers decentralized solution	Data integrity and confidentiality	-	-
Bhat et al. (2022) [58]	-	Mitigates security threats	-	Enhances customer trust
Pelekoudas-Oikonomou et al. (2022) [34]	Offers better life quality	-	Abstain unauthorized access	-

**Table 6:** Summary of studies on the challenges of sharding-based blockchain technology with their main contributions.

Reference	Methodology	Consensus Algorithms	Key Findings
Huang et al. (2022) [65]	Primary quantitative method	Unspecified	Account deployment mechanisms result in inducing imbalanced transaction distribution among the shards, which leads to the issue of hot shards. Results determine that BrokerChain outperforms the solution with a focus on transaction confirmation latency, workload balance, system throughput, and transaction queue size.
Alshahrani et al. (2023) [66]	Secondary method	QoS and Ethereum	Power consumption and scalability are the challenges for integrating sharding-based blockchain technology. Linking computational power and keeping time between block insertions to solve the problem of bitcoin insertion, which results in increased power consumption.
Cai et al. (2024) [67]	Secondary method	Unspecified	IoT blockchain restricts conventional sharding strategies due to their heterogeneous and dynamic nature, leading to the failure of the trade-off between security and throughput. PPO and TPPOD are used for shard reconfiguration when the environment changes.
Antwi et al. (2022) [23]	Primary quantitative method	PBFT, RBFT, WBFT	Scalability issues persist in integrating the IoT within blockchain technology. It also involves the peer-to-peer formation of topology that restricts the achievable throughput.
Florea, Anghel, and Cioara (2022) [51]	Secondary method	IPFS	Interoperability, data personalization, storage, transactional costs, and digital rights management are some challenges.
Yuan et al. (2024) [68]	Secondary method	Unspecified	Due to limited transaction throughput, complex communication requirements, and substantial resource consumption, blockchain technologies face difficulty in managing federated learning tasks. With the help of DAG consensus mechanism, these challenges are addressed and show a 14% improvement in training efficiency.
Huang et al. (2022) [78]	Secondary method	PoS, PoW, CFT, BFT	The increased usage of electronics and home appliances has resulted in increased e-waste affecting the environment. This results in the production of hazardous substances which can be treated by EEE value chain traceability and implementing a circular economy in the value chain.
Zheng et al. (2022) [69]	Secondary method	Unspecified	Blockchain does not provide high-shard efficiency, strict transaction atomicity, and multiple-shard contract calling, resulting in consortium blockchain issues. Through replay epoch, the robustness of the shard is improved, resulting in more than 50,000 TPS under the transaction of real-world shopping behaviors.
Hafid, Hafid, and Makrakis (2023) [53]	Secondary method	PoS	Good performance, good throughput, and reasonable latency and security issues are the challenges.
Jafar et al. (2022) [52]	Secondary method	DSA, RSA, ECC	Scalability is a major barrier. Also, data privacy, verifiability, integrity, transparency, and authentication issues are determined.
Shi, Zeng, and Li (2022) [70]	Secondary method	AP algorithm	Scalability, cybersecurity, and reduced efficiency and reliability issues are the challenges of the technology. It shows an increased performance when large-scale blockchains are used, through increased assurance and real-time processing speed.
Alzoubi and Mishra (2024) [54]	Secondary method	Unspecified	The challenges involve the loss of data, and privacy and security concerns in securing IoT sensors.
Ali and Ahmed (2023) [30]	Secondary method	AGSK	Securing communication is a significant challenge.
Wang and Guan (2022) [44]	Secondary qualitative method	HBFT	Sharding-based networks involve issues in the consistency of transactions with an increased number of nodes. The scalability to assign nodes to various layers results in the improvement in reducing communication difficulties.
Zhao and Ding (2024) [29]	Secondary method	TDGA	Sharding-based blockchain technology results in impacting the overall performance of the network technology.
Valadares et al. (2023) [71]	Secondary method	Byzantine fault-tolerant, Diffie–Hellman	The issues of data security and privacy are more significant in sharding-based blockchain technology. Traceability issues also exist within the blockchain network technology.
Han et al. (2024) [72]	Secondary method	Graph-partitioning algorithm	The problems of scalability and reduced performance result in increased risks of trust management in blockchain. Blockchain technology’s performance is restricted, making it an unsuitable technology for large-scale networks.
Ahakonye, Nwakanma, and Kim (2024) [39]	Secondary method	DDoS, prompting control algorithm	The challenges are cybersecurity, resource constraints and privacy concerns. Privacy-preserving techniques, lightweight cryptography, and efficient consensus mechanisms help deal with issues.
Zubaydi, Varga, and Molnár (2023) [42]	Primary quantitative method	PoA	Scalability issues, the scalability of transaction rates, resource utilization, legal issues, and predictability are the issues.
Adhikari and Ramkumar (2023) [41]	Secondary method	BCN	The significant challenges can lead to scaling, interoperability, and security concerns. The technology involves heterogeneous and dynamic computing, along with communication requirements.
Schmid, Schaffhäuser, and Kashef (2023) [49]	Secondary method	PoW	Challenges include PLC networks, leading to privacy and security concerns.
Ali et al. (2022) [59]	Secondary methods	BSS	The significant challenges include reliability, trustworthiness, confidentiality, and security.
Olanrewaju et al. (2022) [75]	Secondary method	Sophisticated hashing algorithm	Decreased processing time, computational overhead, latency, and energy consumption with increased throughput within the already existing methods. The implementation of security architecture is effective in preventing side-channel attacks.
Huang, Zhao, and Ran (2022) [78]	Secondary method	Unspecified	Blockchain determines insufficient scalability which does not allow it to meet the needs of large-scale applications. Sharding-blockchain maximizes transaction process through VRF, and malicious nodes are controlled to minimize fragmentation deterioration probability.
Wijesekara and Gunawardena (2023) [63]	Secondary method	SIDH, DAG, digital signature algorithm	It determines issues like difficulty in processing large data, increased consumption of energy, and scalability problems. Dissemination and knowledge generation helps resolve these issues.
Musa et al. (2023) [76]	Primary quantitative method	PoW, PoS	The integration of blockchain has drawbacks that involve performance, scalability, complexity, and cost issues. Blockchain-based Android data storage proposes strategies for technical optimization to deal with complexities.
Wu et al. (2024) [77]	Secondary qualitative method,	PQFN	Extended transaction confirmation times pose a challenge due to isolated states between the allocated strategies of shards and unbalanced transactions. This causes an increase in disproportionate shard workloads and cross-shard transactions delay in cross-shard transactions.
Christidis et al. (2022) [73]	Secondary method	Unspecified	Centralized data have to deal with several challenges, which include limited storage and uncertain authenticity.
Hashim, Shuaib, and Sallabi (2022) [38]	Secondary method	PoA	It determines the issue of scalability because of its consensus mechanism and ledger replication.
Ali et al. (2023) [64]	Secondary method	ECC, hybrid encryption framework algorithm	Issues of protection, confidentiality, and data privacy occur within the sharding-based blockchain network technology. The study involved GT-based binary string search algorithms and determined intrusion within the IoT network.

**Table 7:** Comparison between the studies on the challenges of sharding-based blockchain technology.

References	Scalability	Security	Interoperability	Transactional Issues
Huang et al. (2022) [65]	-	-	-	Imbalanced transaction distribution
Alshahrani et al. (2023) [66]	Scalability issues and power consumption	-	-	-
Cai et al. (2024) [67]	-	Failed security and throughput	-	-
Antwi et al. (2022) [23]	Scalability issues	Restricts achieving throughput	-	-
Florea, Anghel, and Cioara (2022) [51]	-	Data personalization	Interoperability issues	Transactional costs
Yuan et al. (2024) [68]	-	-	Complex communication	Limited transaction throughput
Huang et al. (2022) [78]	-	Increased e-waste affects the environment	-	-
Zheng et al. (2022) [69]	-	-	-	Strict transaction atomicity and consortium issues
Hafid, Hafid, and Makrakis (2023) [53]	Good throughput and performance	Security issues and reasonable latency	-	-
Jafar et al. (2022) [52]	Scalability issues	Data integrity, privacy, and transparency	-	-
Shi, Zeng, and Li (2022) [70]	Scalability issues	Cybersecurity issues	Reduced efficiency	-
Alzoubi and Mishra (2024) [54]	-	Data loss, security, and privacy		-
Ali and Ahmed (2023) [30]	-	-	Secured communication	-
Wang and Guan (2022) [44]	-	-	Communication difficulties	Transaction consistency
Zhao and Ding (2024) [29]	Affects performance	-	-	-
Valadares et al. (2023) [71]	-	Data security and privacy	Traceability issues	-
Han et al. (2024) [72]	Reduced performance	-	-	-
Ahakonye, Nwakan-ma, and Kim (2024) [39]	-	Cybersecurity and privacy issues	Resource constraints	-
Zubaydi, Varga, and Molnár (2023) [42]	Scalability and legal issues	-	-	Scalability of transaction rates
Adhikari and Ram Kumar (2023) [41]	Scalability issues	Security concerns	Interoperability concerns	-
Schmid, Schaffhäuser, and Kashef (2023) [49]	-	Privacy and security concerns	-	-
Ali et al. (2022) [59]	-	Security and confidentiality issues	-	-
Olanrewaju et al. (2022) [75]	Latency, computational overhead	Security architecture	-	-
Huang, Zhao, and Ran (2022) [78]	Insufficient scalability	-	-	Maximizes transaction process
Wijesekara and Guna-wardena (2023) [63]	Increased energy consumption and scalability issues	Data processing issues	-	-
Musa et al. (2023) [76]	Scalability and performance issues	-	-	Transactional costs issues
Wu et al. (2024) [77]	-	-	-	Extended transaction confirmation and delay
Christidis et al. (2022) [73]	-	-	Uncertain authenticity and limited storage	-
Hashim, Shuaib, and Sallabi (2022) [38]	Scalability issues, ledger replication	-	-	-
Ali et al. (2023) [74]	-	Data confidentiality and privacy	-	-

## Data Availability

Data is contained within the article.
